# Prognostic Significance of PTTG1 and Its Methylation in Lung Adenocarcinoma

**DOI:** 10.1155/2022/3507436

**Published:** 2022-02-24

**Authors:** Lu Bai, Li-Hong Li, Jing Liang, En-Xiao Li

**Affiliations:** ^1^Department of Medical Oncology, The First Affiliated Hospital of Xi'an Jiaotong University, Xi'an, Shaanxi, China; ^2^Department of Geriatric Respiration, Xi'an No. 1 Hospital, Xi'an, Shaanxi, China

## Abstract

Pituitary tumor-transforming gene-1 (PTTG1), one type of DNA repair-related gene, has been reported to be dysregulated in several tumors and serve as a tumor promotor. Previously, the oncogenic roles of PTTG1 were also reported in lung adenocarcinoma (LUAD). However, the prognostic values of PTTG1 in LUAD and the possible mechanism of its dysregulation have not been clarified. We analyzed TCGA datasets and reported that PTTG1 expression showed a distinct increase within LUAD specimens in comparison with nontumor specimens. Further survival study revealed that patients containing a great PTTG1 level had noticeably less overall survival and progression-free survival as compared with patients containing a low PTTG1 level. Multivariate analyses confirmed that PTTG1 expression was a factor of prognosis that is independent in terms of LUAD patients. Besides, PTTG1 methylation had a negative regulation on PTTG1, so PTTG1 had a high expressing level in LUAD tissues. However, the relation between hypermethylation and overall survival was not demonstrated using TCGA datasets. In addition, we observed that LUAD specimens with advanced stages exhibited a higher level of PTTG1. Finally, the dysregulated genes related to PTTG1 expression were screened, and KEGG assays revealed that the above genes were involved in the p53 signaling pathway, indicating the possible regulatory function of PTTG1 in the p53 signaling pathway. Overall, our findings suggest that PTTG1 may serve as an efficient clinical biomarker and a therapeutic target for patients suffering from LUAD.

## 1. Introduction

Lung carcinoma has been recognized as a highly common malignant tumor worldwide [1]. Lung carcinoma turns out to be the first main factor causing people in China's urban regions to die [2]. Lung adenocarcinoma (LUAD) refers to the commonest among lung carcinoma and has become a popularly aggressive carcinoma [3]. Though LUAD treatment and diagnosis using surgical methods and/or adjuvant chemotherapy have greatly progressed, patients impacted still have poor prognostic results since over 80% patients impacted have the advanced-stage diagnosis [4, 5]. Accordingly, probable prognosis factors should be explored according to survivors for gaining more insights into LUAD malignancy and developing alternatives to treat various subgroups of patients with LUAD.

Pituitary tumor-transforming gene-1 (PTTG1), a ubiquitously expressed factor of transcription, is proven with the overexpression within several types of tumors, including lung carcinoma, ovarian carcinoma, prostate carcinoma, and breast carcinoma [6–8]. In recent years, growing evidence has shown that PTTG1 exhibits regulatory functions in the progression of angiogenesis, DNA damage repair, programmed cell death, and differentiation [9, 10]. In addition, it has also been confirmed that the dysregulation of PTTG1 was related to the abilities of proliferation and metastasis. For instance, PTTG1 expression was distinctly increased in cholangiocarcinoma, and its knockdown suppressed tumor growth via modulating the pathway of MAPK signaling [11]. PTTG1 was also shown with an ability of promoting the invasion of migration of LUAD cells, and its levels were regulated by miR-186, implying a possible role for PTTG1 in LUAD [12]. In addition, the prognostic values of PTTG1 were also reported. However, the studies are limited, and the possible mechanism of PTTG1 dysregulation has not been clarified.

DNA methylation, frequently occurring at CpG dinucleotides, is demonstrated to be related to clinical progression of patients suffering from LUAD, such as TP53 status, carcinoma status, WHO grade, and clinical stages [13, 14]. In recent years, several studies have demonstrated the positive relation between the pattern of single gene and methylation state [15, 16]. However, the specific clinical significance of the methylated markers in LUAD subtypes and the complex role of DNA methylation remained largely unclear, which needed to be further demonstrated in clinical cohorts of patients suffering from LUAD. This paper aimed at exploring the expressing pattern of PTTG1 within patients suffering from LUAD. An analysis was conducted on the relation of PTTG1 DNA methylation and PTTG1 expression within the LUAD dataset of TCGA datasets. Finally, we examined the prognostic significance of PTTG1 expression in LUAD patients and its DNA methylation.

## 2. Materials and Methods

### 2.1. Raw Data

Transcriptome RNA-seq data of 59 normal samples and 526 tumor samples were downloaded from TCGA database (https://portal.gdc.carcinoma.gov/) with level 3. The following samples were excluded: (1) “0” gene expression value and (2) insufficient survival information. A total of 513 patients with LUAD with the corresponding clinical characteristics were enrolled in this paper. Then, we downloaded the methylation profiles of patients with LUAD from TCGA database via UCSC Xena (https://xena.ucsc.edu/).

### 2.2. Relation Assays of PTTG1 Expressions and Methylation of CpG Sites

The relations of PTTG1 expressions with the methylation of CpG sites in different regions of the PTTG1 gene were studied by the use of Pearson's relation tests. The relations of PTTG1 expressions with the methylation of each CpG site were examined.

### 2.3. Relation of PTTG1 CpG Sites with the Characteristics of LUAD

The clinical characteristics of patients suffering from LUAD were extracted, including patients' age, clinical stage, and sex. PTTG1 CpG sites at which methylation states were distinctly related to OS were applied to study their relation with clinical characteristics of LUAD.

### 2.4. GO and KEGG Enrichment Analyses of the Differentially Expressed Genes

Patients with LUAD from TCGA datasets were initially divided into two groups (high and low). The dysregulated genes between the two groups were selected with *p* < 0.05. To study potential biological processes (BP), cellular components (CC), molecular functions (MF), and pathways of the differentially expressed genes, we performed GO and KEGG assays by the use of the “clusterProfiler” package in R with a statistical threshold of *p* < 0.05 [17].

### 2.5. Statistical Analysis

All statistical analyses were based on R language 3.6.1 version. With the use of Fisher's exact test or Pearson chi-squared test, an investigation was conducted on the relation of PTTG1 and clinical feature variables. Kaplan–Meier methods with log-rank tests were applied to determine the overall survival (OS) and progression-free survival (PFS). Significant variables in univariate models were further analyzed by multivariate assays for the identification of independent prognosis factors. *p* ≤ 0.05 was considered to indicate a statistically significant difference.

## 3. Results

### 3.1. The Distinct Upregulation of PTTG1 in LUAD and Its Prognostic Value

**Figure 1:**
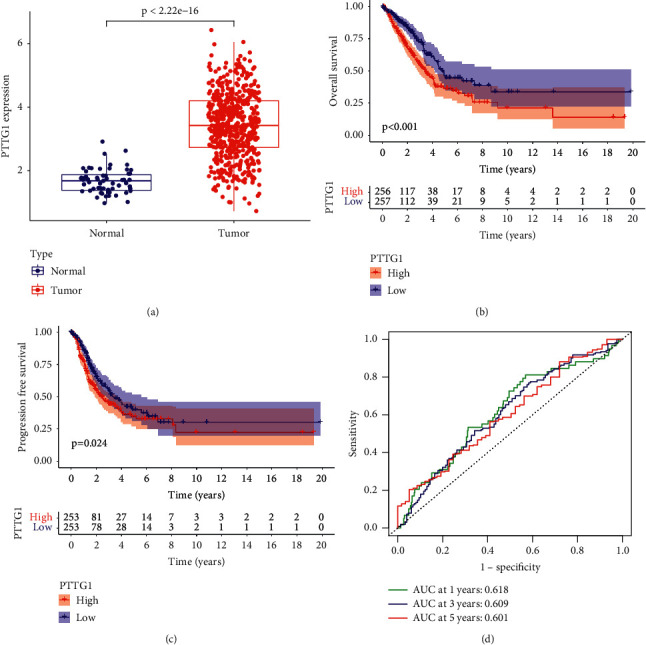
The distinct upregulation of PTTG1 in LUAD patients and its prognostic value. (a) The expression of PTTG1 in LUAD specimens and normal lung specimens from TCGA datasets. (b, c) The overall survival and progression-free survival of LUAD patients based on the expression of PTTG1 in all samples. (d) ROC curve was used to predict the value of PTTG1 expression in predicting the survival of LUAD patients.

**Figure 2:**
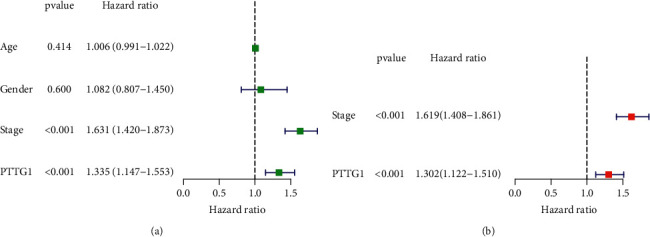
Univariate (a) and multivariate (b) independent prognosis analyses of clinical parameters and PTTG1 expression.

To delve into the potential function of PTTG1 in LUAD, we analyzed TCGA datasets and found that PTTG1 expression was distinctly increased in LUAD specimens in comparison with nontumor lung specimens (Figure 1(a)). We also performed survival assays which revealed that patients with high PTTG1 expression exhibited a shorter OS (*p* < 0.001, Figure 1(b)) and PFS (*p* < 0.024, Figure 1(c)) as compared with those with low PTTG1 expression. The predictive performance of PTTG1 expression for OS was assessed according to time-dependent ROC curves, and the area under the curve (AUC) reached 0.618 at 1 year, 0.609 at 3 years, and 0.601 at 5 years (Figure 1(d)). Moreover, the results of univariate assays revealed that PTTG1 expression and clinical stage were related to OS of patients suffering from LUAD (Figure 2(a)). Further results by multivariate analyses confirmed that PTTG1 expression (HR = 1.302, 95% CI: 1.122–1.510, *p* < 0.001) as well as stage (HR = 1.619, 95% CI: 1.408–1.861, *p* < 0.001) was an independent prognosis factor for the patients with LUAD (Figure 2(b)). Overall, our findings suggested that PTTG1 was an overexpressed gene in LUAD and predicted a poor prognosis of patients suffering from LUAD.

### 3.2. The Relation of DNA Methylation with PTTG1 and Its Survival Analysis

**Figure 3:**
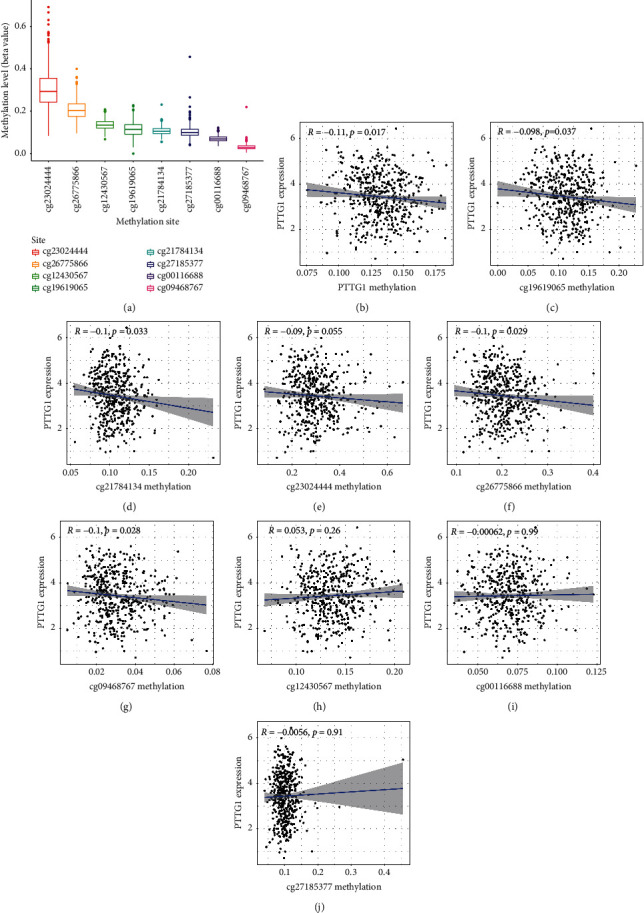
The associations between PTTG1 expressions and methylation of several sites. (a) Histogram of the methylation level in eight methylation sites. (b) The expressions of PTTG1 were negatively modulated by PTTG1 DNA methylation. (c–j) Correlation analysis of PTTG1 with the methylation of (c) cg19619065, (d) cg21784134, (e) cg2302444, (f) cg26775866, (g) cg09468767, (h) cg12430567, (i) cg00116688, and (j) cg27185377.

**Figure 4:**
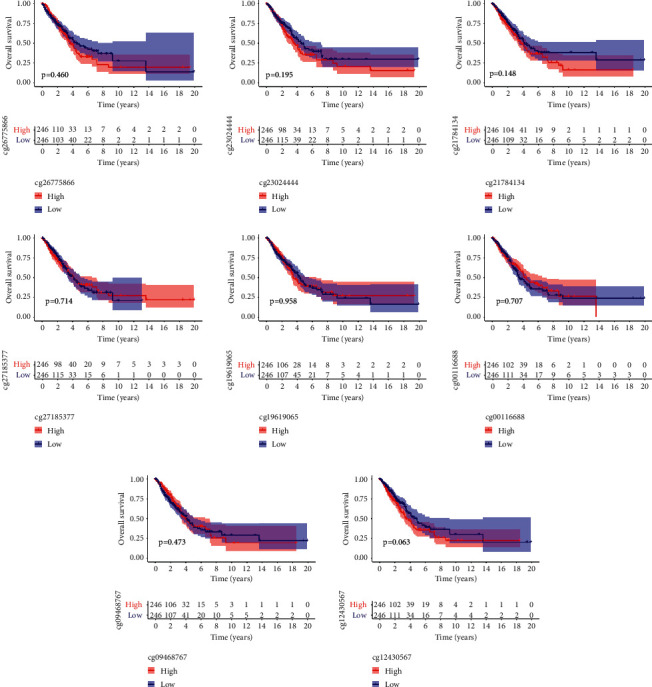
The survival assays of methylation of CpG sites in LUAD patients using Kaplan–Meier methods.

**Figure 5:**
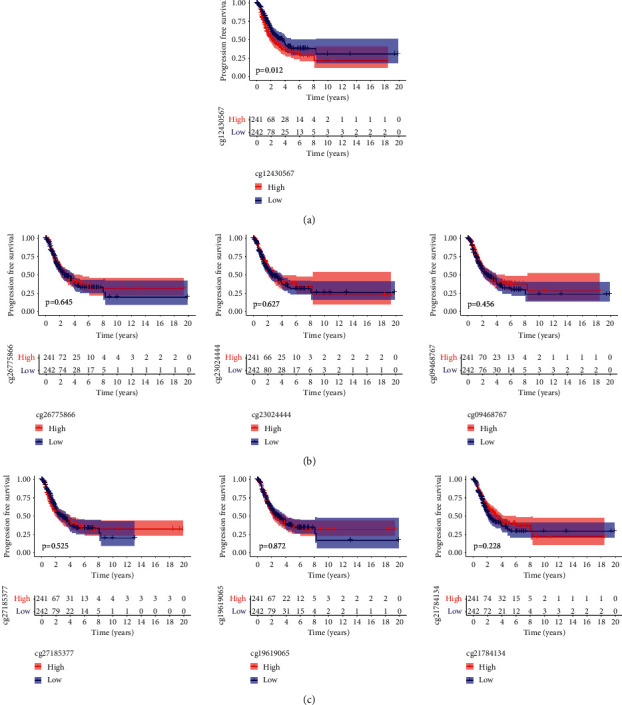
(a) The 5-year progression-free survival rate of LUAD patients with high levels of cg12430567 was distinctly lower than that of those patients with low levels of cg12430567. (b, c) Kaplan–Meier plots of overall survival in patients with LUAD and with low and high levels of methylation sites.

**Figure 6:**
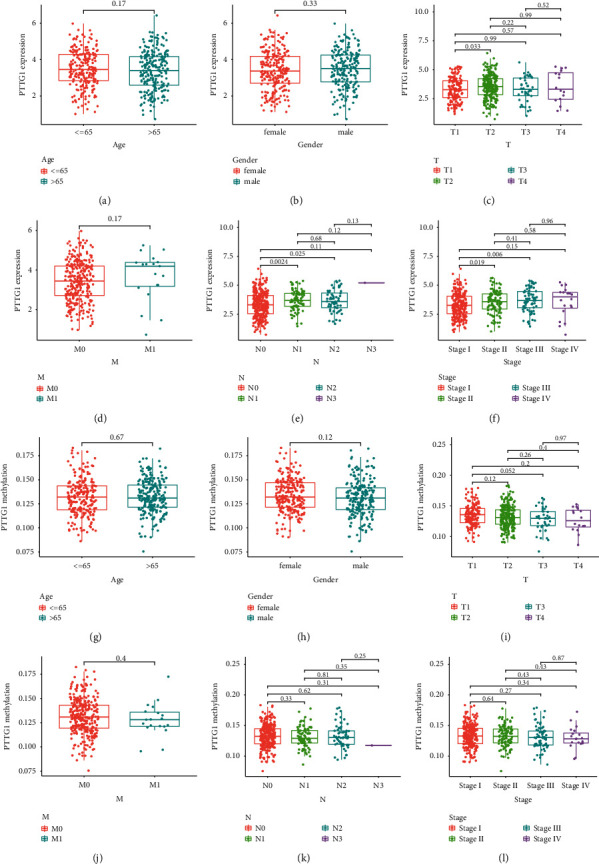
Correlation between PTTG1 expression/methylation and clinicopathologic features in TCGA datasets. (a) Age and PTTG1 expression. (b) Gender and PTTG1 expression. (c) T stage and PTTG1 expression. (d) M stage and PTTG1 expression. (e) N stage and PTTG1 expression. (f) TMN stage and PTTG1 expression. (g) Age and PTTG1 methylation. (h) Gender and PTTG1 methylation. (i) T stage and PTTG1 methylation. (j) M stage and PTTG1 methylation. (k) N stage and PTTG1 methylation. (l) TMN stage and PTTG1 methylation.

Then, we analyzed the level of methylation sites of PTTG1. The distribution of 8 PTTG1 CpG sites is clearly exhibited in Figure 3(a). In addition, a strong negative relation between PTTG1 expressions and PTTG1 DNA methylation was found (Figure 3(b)). Then, Pearson's relation assays were conducted to screen the PTTG1 CpG sites involved in PTTG1 mRNA expressions. We observed that methylation of cg19619065, cg21784134, cg2302444, cg26775866, and cg09468767 was negatively related to the expressions of PTTG1 (Figures 3(c)–3(g)). However, methylation of cg12430567, cg00116688, and cg27185377 was not related to the expression of PTTG1 (Figures 3(h)–3(j)). On the contrary, to explore the prognostic value of methylation of CpG sites, we performed Kaplan–Meier methods and observed that all CpG sites were not related to OS of patients suffering from LUAD from TCGA datasets (Figure 4). However, patients with high methylation of cg12430567 achieved a shorter PFS as compared with those with low methylation of cg12430567 (Figure 5(a)). Other CpG sites showed no relation with PFS of patients suffering from LUAD (Figures 5(b) and 5(c)). The chi-square test was performed for investigating the specific relation of PTTG1 expression and PTTG1 methylation with several clinical characteristics. As shown in Tables S1 and S2, the expression of PTTG1 was closely related to N stage, clinical stage, and PTTG1 methylation. The relation between PTTG1 expression and clinical characteristics is also shown in Figures 6(a)–6(f), and the relation between PTTG1 methylation and clinical characteristics is shown in Figures 6(g)–6(l). Our results indicated that the levels of PTTG1 were modulated by methylation. However, the prognostic value of most CpG sites of PTTG1 was also confirmed.

### 3.3. Functional Analyses of the Dysregulated Genes in TCGA Cohort

**Figure 7:**
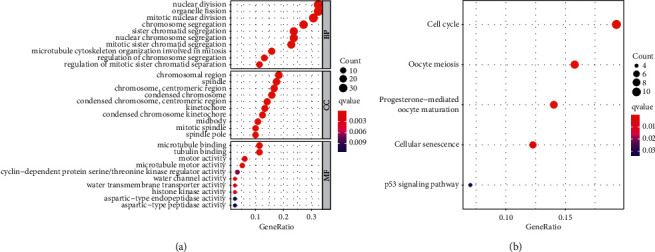
GO (a) and KEGG (b) enrichment analyses of genes associated with PTTG1 expression.

For a clarification of the functional effect of PTTG1 on LUAD, we divided all patients suffering from LUAD into two groups (high and low) based on the mean expression of PTTG1. Then, we screened the dysregulated genes between samples containing high PTTG1 expression and samples with low PTTG1 expression. The dysregulated genes are presented in Table S3. Subsequently, we performed GO assays using the “clusterProfiler” R package and found that, in the BP group, the dysregulated genes were primarily involved in the regulation of mitotic sister chromatid separation, cytoskeleton organization involved in mitosis, nuclear division, mitotic nuclear division, chromosome segregation, and sister chromatid segregation. In the CC, the dysregulated genes were mainly involved in condensed chromosome kinetochore, kinetochore, centromeric region, condensed chromosome, spindle, chromosomal region, midbody, and mitotic spindle. In the MF group, the dysregulated genes were mainly involved in microtubule binding, tubulin binding, motor activity, microtubule motor activity, water channel activity, water transmembrane transporter activity, histone kinase activity, and aspartic-type endopeptidase activity (Figure 7(a)). KEGG analysis showed that the dysregulated genes are mainly enriched in cell cycle, oocyte meiosis, progesterone-mediated oocyte maturation, cellular senescence, and p53 signaling pathway (Figure 7(b)). Our findings suggested that PTTG1 expression was related to progression of LUAD.

## 4. Discussion

New strategies for treatment in terms of LUAD are increasingly designed, which consist of immunotherapy, gene therapy, and molecular targeted therapy [18, 19]. Nevertheless, there were not any satisfactory therapeutic results, and a low survival rate of LUAD has been achieved. New therapeutic and prognostic methods aiming to optimize the outcome of LUAD patients require an overall insight into the molecular mechanism of tumor initiation and progression [20, 21]. Recently, DNA repair-related genes emerge as a novel gene regulator class in various malignancies [22, 23].

As a DNA repair-related gene, the expression and function of PTTG1 have been reported in several tumors. For instance, PTTG1 expression was distinctly increased in glioma, and its knockdown suppressed cell angiogenesis and metastasis in glioma cells [24]. A previous study reported that PTTG1, an overexpressed gene in seminoma tumor, promoted the migration and invasion of tumor cells via activation of MMP-2 [25]. In addition, the prognostic values of PTTG1 were also reported in several tumors, such as breast carcinoma and prostate carcinoma [8, 26]. According to the findings above, PTTG1 is an oncogene in the above tumors. Importantly, Li et al. also reported that PTTG1 was highly expressed in lung carcinoma, and its knockdown distinctly suppressed the invasion and migration of lung carcinoma cells. In their cohort, they also reported upregulation of PTTG1 was related to poor prognosis of patients with lung carcinoma [12]. However, the sample size was small in their cohort. In this paper, we analyzed TGCA datasets and also confirmed that expressions of PTTG1 were distinctly increased in LUAD specimens. Survival assays revealed that patients with high PTTG1 expression showed a shorter OS and PFS as compared with those with low PTTG1 expression. More importantly, in a multivariate Cox model, PTTG1 expression was reported as a poor prognosis factor that is independent in terms of 5-year OS. Clinical stage has been considered to be a very important prognostic factor for LUAD patients, which was also further demonstrated in this study. Thus, whether PTTG1 expression may be associated with clinical stages of LUAD patients needed to be further explored. Overall, our findings suggested PTTG as a novel biomarker for LUAD.

Increasing evidence proved that the dysregulation of DNA methylation significantly impacts the developments and progressions of LUAD [27, 28]. Our group firstly examined if the PTTG1 methylation state could have an effect on PTTG1 expressions by the use of Pearson's coefficients. A potent negative relation of PTTG1 methylation and PTTG1 expressions was found in LUAD tissues. Such a negative relation could effectively account for the high LUAD expression within LUAD tissues. Subsequently, we further screened the specific CpG sites. It is noteworthy that nearly all the CpG sites with the exception of cg27185377 and cg00116688 were obviously related to PTTG1 expressions. In previous studies, the relationship of specific gene expression and its DNA methylation had a range (weak to moderate), and rare genes under the significant regulation by DNA methylation had been found [29, 30]. Furthermore, the prognosis value of PTTG1 DNA methylation and 8 selected CpG sites was explored, and we found that the levels of PTTG1 methylation were not related to the OS and PFS in patients suffering from LUAD. Only cg12430567 was related to OS and patients suffering from LUAD. Our findings suggested PTTG1 was negatively regulated by PTTG1 methylation. However, more experiments were needed to further demonstrate the prognostic value of the PTTG1 methylation state.

There were several limitations in this research. Firstly, the sample size was relatively small, and more clinical experiments were necessary to demonstrate our findings. Secondly, we did not perform in vitro and in vivo experiments to study the potential function of PTTG1 in LUAD progression. Finally, we did not explore the downstream factors which PTTG1 modulated.

## 5. Conclusion

This paper identified PTTG1 hypermethylation state as a prognosis factor in LUAD. Methylation of cg12430567 was related to the survival of patients suffering from LUAD. Our findings indicated the effects of PTTG1 methylation on the pathogenesis of LUAD and provided new targeting genes for predicting the clinical outcomes of patients suffering from LUAD.

## Data Availability

The data used to support the findings of this study are available from the corresponding authors upon request.

## Supplementary Materials

Supplementary Materials Table S1: correlation between PTTG1 expression and clinicopathologic features in TCGA database. Table S2: correlation between PTTG1 methylation and clinicopathologic features in TCGA database. Table S3: the dysregulated genes between the high PTTG1 expression group and low PTTG1 expression group.
