# Supplementary material for: Prognostic Significance of PTTG1 and Its Methylation in Lung Adenocarcinoma
Source: J Oncol. 2022 Feb 24;2022:3507436. doi: 10.1155/2022/3507436 (PMC8894038; doi:10.1155/2022/3507436)
Supplement: Supplementary Materials — Table S1: correlation between PTTG1 expression and clinicopathologic features in TCGA database. Table S2: correlation between PTTG1 methylation and clinicopathologic features in TCGA database. Table S3: the dysregulated genes between the high PTTG1 expression group and low PTTG1 expression group. [file 3507436.f1.zip › 3507436.f1/Table S1 (1).docx]

**Table S1** Correlation between PTTG1 expression and clinicopathologic features in TCGA database.

| Covariates | Type | Total | High | Low | Pvalue |
| --- | --- | --- | --- | --- | --- |
| Age | <=65 | 222(48.26%) | 115(50%) | 107(46.52%) | 0.5137 |
|  | >65 | 238(51.74%) | 115(50%) | 123(53.48%) |  |
| M stage | M0 | 299(65%) | 151(65.65%) | 148(64.35%) | 0.4045 |
|  | M1 | 19(4.13%) | 12(5.22%) | 7(3.04%) |  |
|  | unknow | 142(30.87%) | 67(29.13%) | 75(32.61%) |  |
| N stage | N0 | 305(66.3%) | 138(60%) | 167(72.61%) | 0.0098 |
|  | N1 | 81(17.61%) | 51(22.17%) | 30(13.04%) |  |
|  | N2 | 64(13.91%) | 38(16.52%) | 26(11.3%) |  |
|  | N3 | 1(0.22%) | 1(0.43%) | 0(0%) |  |
|  | unknow | 9(1.96%) | 2(0.87%) | 7(3.04%) |  |
| T stage | T1 | 55(11.96%) | 26(11.3%) | 29(12.61%) | 0.2929 |
|  | T1a | 46(10%) | 19(8.26%) | 27(11.74%) |  |
|  | T1b | 54(11.74%) | 23(10%) | 31(13.48%) |  |
|  | T2 | 244(53.04%) | 135(58.7%) | 109(47.39%) |  |
|  | T3 | 42(9.13%) | 19(8.26%) | 23(10%) |  |
|  | T4 | 16(3.48%) | 7(3.04%) | 9(3.91%) |  |
|  | unknow | 3(0.65%) | 1(0.43%) | 2(0.87%) |  |
| Gender | female | 245(53.26%) | 118(51.3%) | 127(55.22%) | 0.4547 |
|  | male | 215(46.74%) | 112(48.7%) | 103(44.78%) |  |
| Stage | Stage I | 253(55%) | 110(47.83%) | 143(62.17%) | 0.0171 |
|  | Stage II | 111(24.13%) | 62(26.96%) | 49(21.3%) |  |
|  | Stage III | 72(15.65%) | 44(19.13%) | 28(12.17%) |  |
|  | Stage IV | 20(4.35%) | 12(5.22%) | 8(3.48%) |  |
|  | unknow | 4(0.87%) | 2(0.87%) | 2(0.87%) |  |
| PTTG1 expression | High | 230(50%) | 230(100%) | 0(0%) | 0 |
|  | Low | 230(50%) | 0(0%) | 230(100%) |  |
| Methylation | High | 230(50%) | 103(44.78%) | 127(55.22%) | 0.032 |
|  | Low | 230(50%) | 127(55.22%) | 103(44.78%) |  |
