# Supplementary material for: Prognostic Significance of PTTG1 and Its Methylation in Lung Adenocarcinoma
Source: J Oncol. 2022 Feb 24;2022:3507436. doi: 10.1155/2022/3507436 (PMC8894038; doi:10.1155/2022/3507436)
Supplement: Supplementary Materials — Table S1: correlation between PTTG1 expression and clinicopathologic features in TCGA database. Table S2: correlation between PTTG1 methylation and clinicopathologic features in TCGA database. Table S3: the dysregulated genes between the high PTTG1 expression group and low PTTG1 expression group. [file 3507436.f1.zip › 3507436.f1/Table S2 (1).docx]

**Table S2** Correlation between PTTG1 methylation and clinicopathologic features in TCGA database.

| Covariates | Type | Total | High | Low | Pvalue |
| --- | --- | --- | --- | --- | --- |
| Age | <=65 | 222(48.26%) | 114(49.57%) | 108(46.96%) | 0.6408 |
|  | >65 | 238(51.74%) | 116(50.43%) | 122(53.04%) |  |
| M stage | M0 | 299(65%) | 143(62.17%) | 156(67.83%) | 0.4883 |
|  | M1 | 19(4.13%) | 7(3.04%) | 12(5.22%) |  |
|  | unknow | 142(30.87%) | 80(34.78%) | 62(26.96%) |  |
| N stage | N0 | 305(66.3%) | 159(69.13%) | 146(63.48%) | 0.3707 |
|  | N1 | 81(17.61%) | 37(16.09%) | 44(19.13%) |  |
|  | N2 | 64(13.91%) | 28(12.17%) | 36(15.65%) |  |
|  | N3 | 1(0.22%) | 0(0%) | 1(0.43%) |  |
|  | unknow | 9(1.96%) | 6(2.61%) | 3(1.3%) |  |
| T stage | T1 | 55(11.96%) | 30(13.04%) | 25(10.87%) | 0.1394 |
|  | T1a | 46(10%) | 24(10.43%) | 22(9.57%) |  |
|  | T1b | 54(11.74%) | 35(15.22%) | 19(8.26%) |  |
|  | T2 | 244(53.04%) | 117(50.87%) | 127(55.22%) |  |
|  | T3 | 42(9.13%) | 16(6.96%) | 26(11.3%) |  |
|  | T4 | 16(3.48%) | 7(3.04%) | 9(3.91%) |  |
|  | unknow | 3(0.65%) | 1(0.43%) | 2(0.87%) |  |
| Gender | female | 245(53.26%) | 127(55.22%) | 118(51.3%) | 0.4547 |
|  | male | 215(46.74%) | 103(44.78%) | 112(48.7%) |  |
| Stage | Stage I | 253(55%) | 134(58.26%) | 119(51.74%) | 0.2884 |
|  | Stage II | 111(24.13%) | 57(24.78%) | 54(23.48%) |  |
|  | Stage III | 72(15.65%) | 30(13.04%) | 42(18.26%) |  |
|  | Stage IV | 20(4.35%) | 8(3.48%) | 12(5.22%) |  |
|  | unknow | 4(0.87%) | 1(0.43%) | 3(1.3%) |  |
| PTTG1 expression | High | 230(50%) | 103(44.78%) | 127(55.22%) | 0.032 |
| PTTG1 expression | Low | 230(50%) | 127(55.22%) | 103(44.78%) |  |
| Methylation | High | 230(50%) | 230(100%) | 0(0%) | 0 |
| Methylation | Low | 230(50%) | 0(0%) | 230(100%) |  |
