# Supplementary material for: Prognostic Significance of PTTG1 and Its Methylation in Lung Adenocarcinoma
Source: J Oncol. 2022 Feb 24;2022:3507436. doi: 10.1155/2022/3507436 (PMC8894038; doi:10.1155/2022/3507436)
Supplement: Supplementary Materials — Table S1: correlation between PTTG1 expression and clinicopathologic features in TCGA database. Table S2: correlation between PTTG1 methylation and clinicopathologic features in TCGA database. Table S3: the dysregulated genes between the high PTTG1 expression group and low PTTG1 expression group. [file 3507436.f1.zip › 3507436.f1/Table S3 (1).docx]

Table S3 The dysregulated genes between high PTTG1 expression group and low PTTG1 expression group.

| gene | conMean | treatMean | logFC | pValue | fdr |
| --- | --- | --- | --- | --- | --- |
| NCAPG | 1.260593 | 2.382856 | 1.122263 | 3.47E-48 | 6.07E-45 |
| TNNT1 | 1.870146 | 2.875971 | 1.005825 | 3.58E-10 | 3.55E-09 |
| WIF1 | 2.562532 | 1.428913 | -1.13362 | 1.17E-10 | 1.28E-09 |
| NEK2 | 1.728572 | 2.923336 | 1.194764 | 1.02E-40 | 4.23E-38 |
| PBK | 1.524196 | 2.695337 | 1.171141 | 6.08E-37 | 1.55E-34 |
| DLGAP5 | 1.613878 | 2.815247 | 1.201368 | 4.27E-43 | 2.75E-40 |
| RAP1GAP | 4.102998 | 3.068143 | -1.03485 | 2.17E-17 | 7.39E-16 |
| SCGB3A1 | 6.289267 | 4.44964 | -1.83963 | 4.14E-12 | 5.90E-11 |
| ELN | 4.1941 | 2.974603 | -1.2195 | 2.01E-25 | 1.81E-23 |
| TOP2A | 3.258417 | 4.629547 | 1.371129 | 5.32E-36 | 1.24E-33 |
| ASF1B | 2.461975 | 3.583939 | 1.121964 | 6.14E-47 | 7.91E-44 |
| CENPA | 1.235902 | 2.445461 | 1.209559 | 2.38E-48 | 5.29E-45 |
| SFTPA2 | 8.820949 | 7.488253 | -1.3327 | 2.16E-05 | 7.98E-05 |
| SFTPD | 6.568613 | 5.024197 | -1.54442 | 1.88E-13 | 3.38E-12 |
| SLC22A31 | 5.147843 | 3.982599 | -1.16524 | 1.11E-11 | 1.46E-10 |
| FGB | 1.618777 | 2.71079 | 1.092013 | 0.000421 | 0.001135 |
| CDC45 | 1.473062 | 2.571701 | 1.098639 | 6.87E-44 | 5.10E-41 |
| TPX2 | 2.865042 | 4.405605 | 1.540563 | 1.52E-43 | 1.00E-40 |
| CLDN18 | 3.075107 | 1.766718 | -1.30839 | 2.59E-11 | 3.19E-10 |
| NDC80 | 1.382506 | 2.42637 | 1.043864 | 3.05E-42 | 1.70E-39 |
| AQP3 | 7.13692 | 6.067035 | -1.06988 | 5.17E-11 | 6.03E-10 |
| AQP4 | 3.619845 | 2.289133 | -1.33071 | 9.20E-17 | 2.84E-15 |
| PLK1 | 1.923322 | 3.019963 | 1.096641 | 1.37E-40 | 5.48E-38 |
| GKN2 | 2.064165 | 0.820605 | -1.24356 | 1.21E-14 | 2.63E-13 |
| ADH1B | 2.941284 | 1.483258 | -1.45803 | 9.03E-26 | 8.47E-24 |
| MYH11 | 2.95384 | 1.900011 | -1.05383 | 1.17E-23 | 9.03E-22 |
| ZWINT | 3.03435 | 4.178783 | 1.144433 | 8.71E-43 | 5.20E-40 |
| MSLN | 6.126634 | 4.43822 | -1.68841 | 6.63E-11 | 7.59E-10 |
| SLC44A4 | 5.468012 | 4.429336 | -1.03868 | 1.11E-17 | 3.94E-16 |
| RRM2 | 2.555253 | 3.896149 | 1.340896 | 1.59E-42 | 9.25E-40 |
| SLC34A2 | 8.470355 | 7.342565 | -1.12779 | 6.33E-11 | 7.31E-10 |
| TK1 | 4.097911 | 5.363103 | 1.265192 | 5.60E-36 | 1.28E-33 |
| CDC20 | 2.946676 | 4.577259 | 1.630583 | 2.24E-49 | 6.09E-46 |
| KIF4A | 1.587096 | 2.767767 | 1.180671 | 1.59E-41 | 7.93E-39 |
| SELENBP1 | 5.837239 | 4.7177 | -1.11954 | 1.17E-19 | 5.55E-18 |
| PRR11 | 1.646111 | 2.732776 | 1.086666 | 1.70E-40 | 6.58E-38 |
| MKI67 | 2.005238 | 3.084433 | 1.079195 | 1.74E-32 | 3.02E-30 |
| MAD2L1 | 1.458542 | 2.460863 | 1.002321 | 1.98E-42 | 1.13E-39 |
| HJURP | 1.497094 | 2.707684 | 1.21059 | 6.87E-43 | 4.20E-40 |
| MYBL2 | 2.867621 | 4.590167 | 1.722546 | 1.58E-40 | 6.24E-38 |
| EIF4EBP1 | 4.429407 | 5.501432 | 1.072025 | 9.02E-30 | 1.25E-27 |
| CKS2 | 4.614924 | 5.857468 | 1.242544 | 2.34E-47 | 3.18E-44 |
| KIF2C | 1.94328 | 3.244733 | 1.301453 | 6.82E-44 | 5.10E-41 |
| PCLAF | 1.801366 | 2.810958 | 1.009592 | 3.31E-40 | 1.21E-37 |
| INMT | 3.180997 | 1.988307 | -1.19269 | 5.92E-25 | 5.08E-23 |
| CCNA2 | 2.151037 | 3.411063 | 1.260027 | 2.85E-45 | 3.17E-42 |
| FGFR3 | 2.99185 | 1.962137 | -1.02971 | 1.83E-16 | 5.43E-15 |
| TRIP13 | 1.991088 | 3.089705 | 1.098617 | 1.78E-32 | 3.07E-30 |
| PIGR | 6.593613 | 5.113086 | -1.48053 | 1.13E-09 | 1.01E-08 |
| AC099850.3 | 1.980656 | 3.10814 | 1.127484 | 2.02E-35 | 4.49E-33 |
| PGC | 5.616102 | 3.361585 | -2.25452 | 1.71E-12 | 2.61E-11 |
| KIF11 | 2.005233 | 3.010391 | 1.005158 | 8.19E-39 | 2.54E-36 |
| ATP13A4 | 2.834145 | 1.711135 | -1.12301 | 5.72E-19 | 2.45E-17 |
| CEACAM6 | 8.873751 | 7.728569 | -1.14518 | 1.00E-09 | 9.07E-09 |
| NAPSA | 8.383449 | 6.871794 | -1.51165 | 6.21E-14 | 1.22E-12 |
| ALPL | 4.767748 | 3.699874 | -1.06787 | 1.00E-10 | 1.11E-09 |
| SLC26A9 | 3.059237 | 1.883833 | -1.1754 | 2.86E-16 | 8.19E-15 |
| ANLN | 2.191723 | 3.37417 | 1.182448 | 9.79E-31 | 1.44E-28 |
| UBE2C | 3.428447 | 5.246325 | 1.817879 | 5.81E-44 | 4.74E-41 |
| CDCA5 | 1.889268 | 3.136097 | 1.246829 | 3.07E-45 | 3.26E-42 |
| CACNA2D2 | 3.201835 | 1.833651 | -1.36818 | 1.12E-20 | 6.28E-19 |
| CENPW | 2.413059 | 3.670623 | 1.257564 | 2.91E-48 | 5.71E-45 |
| KPNA2 | 4.367515 | 5.479545 | 1.11203 | 9.44E-41 | 3.98E-38 |
| MELK | 1.708329 | 2.948098 | 1.239769 | 7.93E-42 | 4.04E-39 |
| FOXM1 | 2.067663 | 3.330345 | 1.262682 | 2.84E-38 | 8.48E-36 |
| AURKA | 2.324626 | 3.491237 | 1.166611 | 3.49E-40 | 1.25E-37 |
| HMMR | 1.500276 | 2.624134 | 1.123858 | 3.75E-48 | 6.11E-45 |
| TROAP | 1.297607 | 2.537267 | 1.23966 | 1.76E-46 | 2.15E-43 |
| CDKN3 | 1.64729 | 2.954735 | 1.307444 | 5.39E-51 | 2.20E-47 |
| SCGB1A1 | 5.409822 | 3.758403 | -1.65142 | 3.30E-08 | 2.23E-07 |
| CDT1 | 1.804825 | 2.931067 | 1.126242 | 8.60E-44 | 6.19E-41 |
| PTTG1 | 2.592748 | 4.264701 | 1.671953 | 1.27E-87 | 3.11E-83 |
| CCNB2 | 2.161747 | 3.396249 | 1.234502 | 3.04E-48 | 5.71E-45 |
| NCAPH | 1.632132 | 2.723576 | 1.091444 | 1.07E-40 | 4.36E-38 |
| C4BPA | 5.805956 | 4.613272 | -1.19268 | 1.15E-09 | 1.03E-08 |
| SLC22A3 | 3.440815 | 2.352201 | -1.08861 | 3.68E-19 | 1.63E-17 |
| C7 | 4.04986 | 2.694772 | -1.35509 | 1.48E-21 | 9.27E-20 |
| NUSAP1 | 2.74318 | 3.878975 | 1.135795 | 3.36E-44 | 2.94E-41 |
| SCGB3A2 | 6.837145 | 4.584059 | -2.25309 | 3.75E-17 | 1.23E-15 |
| CDK1 | 2.379373 | 3.645189 | 1.265816 | 4.00E-46 | 4.66E-43 |
| CYP4B1 | 4.471563 | 2.511016 | -1.96055 | 4.43E-26 | 4.26E-24 |
| GINS2 | 1.500663 | 2.543025 | 1.042362 | 1.97E-47 | 2.83E-44 |
| PIMREG | 1.017833 | 2.03239 | 1.014557 | 3.05E-44 | 2.87E-41 |
| GGTLC1 | 3.142102 | 1.896148 | -1.24595 | 2.60E-16 | 7.47E-15 |
| CDCA8 | 2.32419 | 3.511917 | 1.187727 | 9.54E-44 | 6.67E-41 |
| FOLR1 | 6.285983 | 4.699253 | -1.58673 | 3.60E-18 | 1.37E-16 |
| KIFC1 | 2.213995 | 3.429429 | 1.215433 | 2.71E-40 | 1.00E-37 |
| NUF2 | 1.435267 | 2.601622 | 1.166356 | 3.23E-44 | 2.93E-41 |
| C16orf89 | 6.306285 | 4.418722 | -1.88756 | 1.60E-20 | 8.74E-19 |
| CEP55 | 2.125314 | 3.366296 | 1.240982 | 3.25E-42 | 1.77E-39 |
| BUB1 | 1.610321 | 2.664871 | 1.05455 | 2.13E-40 | 8.16E-38 |
| UBE2T | 3.312763 | 4.581861 | 1.269098 | 4.02E-44 | 3.39E-41 |
| KIF20A | 1.788481 | 2.97811 | 1.189629 | 5.86E-54 | 3.59E-50 |
| SUSD2 | 4.74749 | 3.043497 | -1.70399 | 3.79E-23 | 2.77E-21 |
| SCTR | 2.58623 | 1.573347 | -1.01288 | 3.63E-16 | 1.01E-14 |
| KIF18B | 1.196153 | 2.241043 | 1.04489 | 3.11E-38 | 9.16E-36 |
| AQP5 | 3.765703 | 2.616687 | -1.14902 | 1.13E-07 | 6.82E-07 |
| MFAP4 | 5.422365 | 4.193481 | -1.22888 | 1.30E-22 | 9.01E-21 |
| PEBP4 | 3.377216 | 2.100164 | -1.27705 | 6.83E-15 | 1.54E-13 |
| MCM2 | 2.887875 | 3.894998 | 1.007123 | 1.54E-35 | 3.45E-33 |
| AGER | 4.284469 | 3.156046 | -1.12842 | 5.21E-10 | 5.00E-09 |
| SFTPC | 6.061472 | 4.191292 | -1.87018 | 2.19E-09 | 1.85E-08 |
| TMPRSS2 | 4.423102 | 3.273772 | -1.14933 | 1.50E-24 | 1.24E-22 |
| SPAG5 | 2.032175 | 3.05824 | 1.026065 | 1.77E-36 | 4.32E-34 |
| CYP2B7P | 4.788028 | 3.24023 | -1.5478 | 3.05E-18 | 1.17E-16 |
| ADGRF5 | 5.300636 | 3.954113 | -1.34652 | 4.52E-25 | 3.91E-23 |
| PRC1 | 2.104224 | 3.127834 | 1.02361 | 1.69E-39 | 5.55E-37 |
| SFTA1P | 4.24105 | 2.865878 | -1.37517 | 2.26E-18 | 8.87E-17 |
| BIRC5 | 2.363704 | 3.941982 | 1.578278 | 5.57E-50 | 1.70E-46 |
| TNS1 | 4.377646 | 3.374719 | -1.00293 | 3.43E-35 | 7.42E-33 |
| NDNF | 4.306457 | 3.172583 | -1.13387 | 6.32E-17 | 2.00E-15 |
| AGR3 | 5.353583 | 4.161294 | -1.19229 | 3.88E-11 | 4.63E-10 |
| VSIG2 | 3.856197 | 2.585644 | -1.27055 | 1.99E-16 | 5.87E-15 |
| MS4A15 | 2.321133 | 1.228584 | -1.09255 | 7.37E-13 | 1.20E-11 |
| UBE2S | 2.221667 | 3.281162 | 1.059495 | 1.18E-39 | 3.96E-37 |
| CTSE | 5.80637 | 4.304524 | -1.50185 | 3.35E-11 | 4.04E-10 |
| C1orf116 | 5.542496 | 4.101892 | -1.4406 | 5.65E-26 | 5.38E-24 |
| SFTA2 | 7.45517 | 6.399996 | -1.05517 | 1.73E-07 | 1.00E-06 |
| MMP12 | 2.560708 | 3.887858 | 1.32715 | 1.85E-16 | 5.47E-15 |
| AURKB | 1.828324 | 3.253524 | 1.4252 | 3.38E-51 | 1.65E-47 |
| SFTPA1 | 8.572332 | 7.183653 | -1.38868 | 7.73E-06 | 3.15E-05 |
| CCNB1 | 3.075659 | 4.516882 | 1.441224 | 5.13E-54 | 3.59E-50 |
| SFTPB | 10.45251 | 8.648583 | -1.80393 | 1.36E-14 | 2.93E-13 |
